# Crickets alter wind-elicited escape strategies depending on acoustic context

**DOI:** 10.1038/s41598-017-15276-x

**Published:** 2017-11-09

**Authors:** Matasaburo Fukutomi, Hiroto Ogawa

**Affiliations:** 1 Graduate School of Life Science, Hokkaido University, Sapporo, 060-0810 Japan; 2 Department of Biological Sciences, Faculty of Science, Hokkaido University, Sapporo, 060-0810 Japan

## Abstract

Acoustic signals trigger various behaviours in insects such as courtship or escape from predators. However, it remains unknown whether insects utilize acoustic signals to recognize environmental contexts. The cricket is a prominent model insect for neuroethological studies on acoustic behaviour because female crickets exhibit positive phonotaxis in response to male calling songs, and flying crickets display avoidance behaviour for high-frequency sounds such as echolocation call of bats. The carrier frequency of these sounds is a major factor in determining whether they initiate these acoustic behaviours. Here, we examined the impacts of different frequencies of tone sounds on cercal-mediated escape behaviour, using a 5-kHz tone corresponding to the calling song and a 15-kHz tone serving as a trigger of avoidance behaviours. Neither frequency elicited a response in the standing cricket by itself, but they had different impacts on walking responses to airflow stimuli. While the 15-kHz tone reduced response probability, extended moving distance, and enhanced turn-angle variability, the 5-kHz tone had no effect. Although both frequencies of tones facilitated walking backward, the 15-kHz tone had a larger effect than the 5-kHz tone. These frequency dependencies of behavioural modulation suggest that crickets can recognize acoustic contexts and alter their escape strategy accordingly.

## Introduction

Audition is one of the essential sensory modalities used to sense environments and direct appropriate behaviours. Similar to vertebrates, insects detect various auditory cues to serve in adaptive decision making. For mating-related communication in several insect species, male insects use acoustic signals (i.e. calling songs) to attract conspecific female insects^1–3^. Meanwhile, most nocturnal flying insects detect the ultrasonic echolocation calls of bats and exhibit avoidance behaviours in response^4–6^. These acoustic signals are considered trigger stimuli which induce stereotyped behaviours^7,8^. In vertebrates, the acoustic signals which trigger no response alone can provide ‘acoustic context’ used for behavioural modulation when adapting to environmental conditions^9,10^. However, in insects, it remains unclear whether audible but non-triggering stimuli can modulate the behaviour elicited by other sensory inputs.

The cricket has been used as a prominent model animal for neuroethological studies of acoustic behaviour^11^. Carrier frequency of sound is a major factor of acoustic context for crickets. During flight, crickets exhibit two distinct behaviours depending on the sound frequency: first, a positive steering induced by a conspecific male cricket’s calling song, of which the carrier frequency is ~5 kHz; second, an avoidance reflex in response to a high-frequency sound imitating a foraging bat's echolocation call (>10 kHz); these behaviours are known as positive and negative phonotaxis, respectively^12–15^. Two ascending auditory neurons, identified as AN1 and AN2, within the prothoracic ganglion project their axons to the brain^16–18^. AN1 and AN2 are sensitive to different frequency ranges: AN1 is tuned to a low frequency corresponding to the calling song, while AN2 responds mainly to a higher frequency spanning the echolocation calls^16–18^. These neurons are involved in positive and negative phonotaxis, respectively. For positive phonotaxis, AN1 conveys information about the temporal pattern of a conspecific's calling song to a neural circuit within the brain^17,19,20^. Firing activity of AN2 encodes the information of high-frequency sound and triggers inflection of the cricket's abdomen during flight, one of the negative phonotaxis behaviours^5^. These evidences demonstrate that the crickets can distinguish acoustic contexts by hearing the difference in sound frequency. On the ground, however, female crickets approach the sound source of the calling song, but exhibit no behavioural response to the high-frequency sound like the bat echolocation call, which should be heard by the crickets^21,22^. In addition, firing activities of AN1 or AN2 evoked by pure tone sounds elicit no specific behaviour in the standing crickets^17,23^. On the other hand, the cricket exhibits an escape response to a short air-puff detected by cerci, which is considered as a defensive behaviour against lunging predators such as spider^24,25^. In a previous study, we explored the auditory impact on the wind-elicited escape behaviour and reported that a preceding acoustic stimulus of a 10-kHz pure tone that evoked no response alone modulated moving direction and response threshold of wind-elicited walking^26^. This fact revealed that a cross-modal interaction between auditory and cercal sensory systems caused behavioural changes in escape strategy, suggesting that crickets perceived the acoustic signals and interpreted them as acoustic contexts. However, it was unclear whether the crickets used sound-frequency information representing distinct contexts for the modulation of the wind-elicited escape behaviour.

To address this issue, we adopted the frequency-dependent acoustic context to test the behavioural framework of auditory modulation of wind-elicited escape behaviour in the cricket^26^. We applied two different frequencies of sound (a 5- or 15-kHz pure tone), which were initiated preceding an air puff and terminated simultaneously, and we measured wind-elicited escape walking using a spherical treadmill system. We found that the high-frequency sound decreased response probability, increased walking distance, and expanded variance of turn, but the low-frequency sound had no effect. In addition, both frequencies of sounds facilitated walking in the backward direction, but the backward effect induced by the high-frequency tone was larger in angular magnitude than that of the low-frequency tone. These differences in behavioural modulations between the two frequencies of sounds suggest that crickets can adapt their escape strategy depending on the acoustic context.

## Results

### Frequency dependency of the auditory modulation of locomotor activities in wind-elicited escape walking

**Figure 1:**
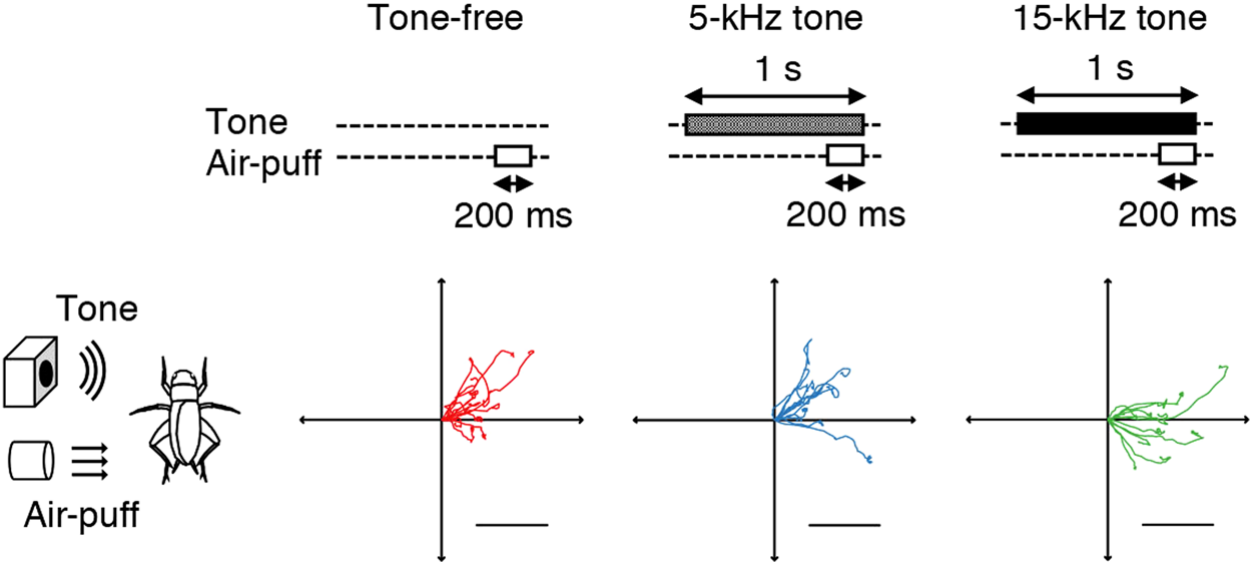
Experimental designs and typical walking trajectories of initial responses to air puffs combined with tone sounds of different frequencies. Upper diagrams show the temporal arrangements of auditory and air-puff stimuli. Three types of stimuli were randomly applied to an individual cricket. Left diagrams show the spatial arrangements of the stimulation. Colour traces show typical walking trajectories in response to different types of stimuli in an individual. Scale bars indicate 10 mm on the virtual plane.

**Figure 2:**
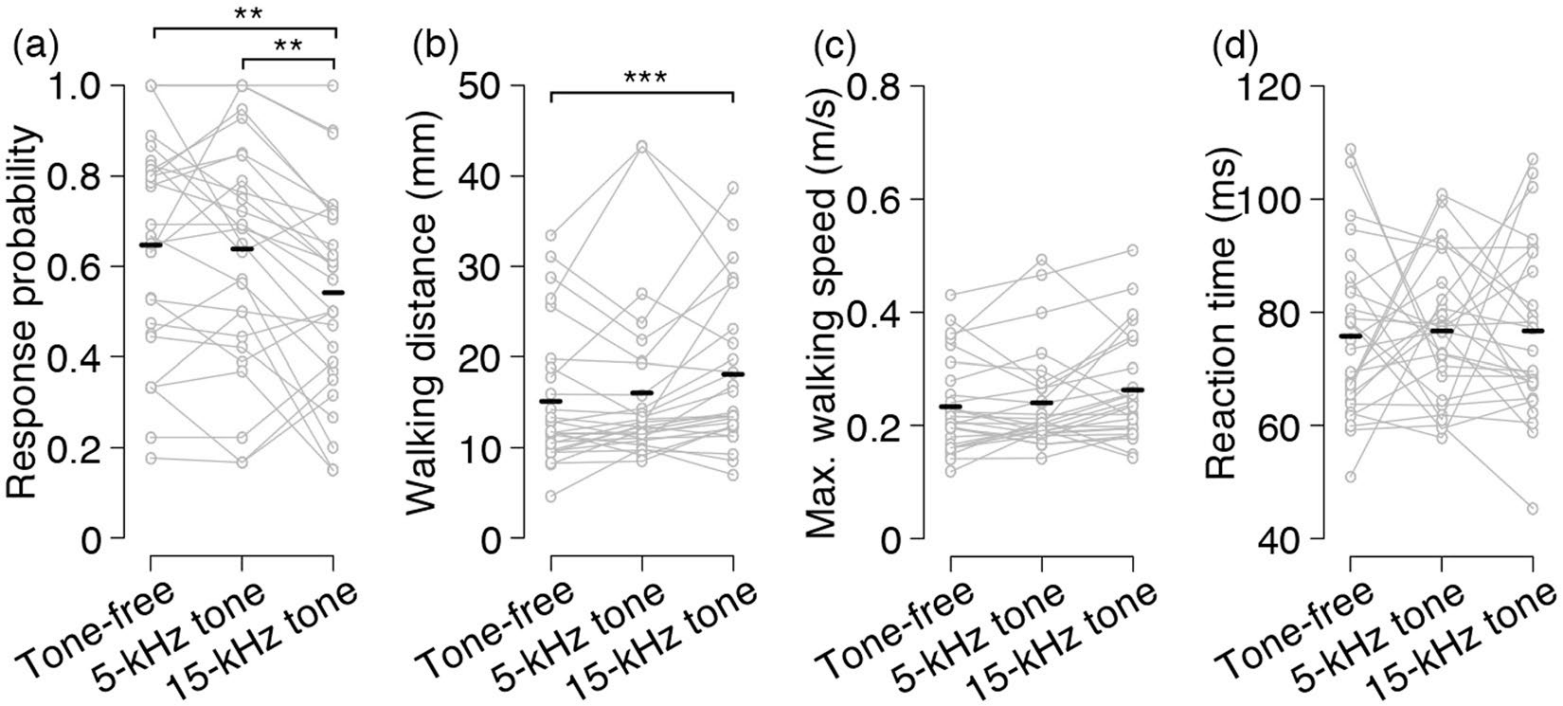
Auditory effects of different frequencies of tones on the wind-elicited escape. Four different locomotion parameters including response probability (**a**), mean walking distance (**b**), maximum walking speed (**c**), and reaction time (**d**) were compared among the three types of stimulation. Each black bar indicates average value across the population for each type of stimulation, and grey circles denote individuals (N = 27 individuals). ***p < 0.001, **p < 0.01 (paired t-test or Wilcoxon paired-sample test followed by Holm’s correction).

Firstly, we examined the carrier-frequency dependence on the cross-modal impacts of the sound on the escape response to air-puff stimulus from the lateral side (Fig. 1). Our previous study revealed that a 10-kHz tone elevated the response threshold of the escape walking elicited by the lateral stimulus^26^. In this study, we observed significant effects of a sound on the wind-elicited response probability and walking distance, and these effects depended on the sound frequency (Fig. 2a and b). The 15-kHz tone reduced response probability, but 5-kHz tone did not affect it (*p* = 0.0073 comparing tone-free and 15-kHz tone protocols, *p* = 0.7590 comparing tone-free and 5-kHz tone protocols, *p* = 0.0073 comparing 5-kHz tone and 15-kHz tone protocols, paired t-tests followed by Holm’s correction). In addition, a 15-kHz tone increased walking distance in the initial response whereas 5-kHz tone did not change it (*p* = 0.0006 comparing tone-free and 15-kHz tone protocols, *p* = 0.4698 comparing tone-free and 5-kHz tone, *p* = 0.0982 comparing 5-kHz tone and 15-kHz tone protocols, Wilcoxon paired-sample test followed by Holm’s correction). In contrast, there was no significant difference among the stimulation types in maximum walking speed (*p* = 0.0449, one-way repeated-measures ANOVA, *p* = 0.0954 for tone-free vs 15-kHz tone, *p* = 0.4477 for tone-free vs 5-kHz tone, *p* = 0.2031 for 5-kHz tone vs 15-kHz tone protocols, Holm’s corrected paired t-tests) (Fig. 2c) and reaction time (*p* = 0.9572, one-way repeated-measures ANOVA) (Fig. 2d). Taken together, high-frequency sound decreased the responsiveness to the air puff but extended the escaping distance.

### Frequency dependency of the auditory modulation of walking direction

In our previous study, an auditory stimulus (10-kHz pure tone) facilitated walking in the backward direction in response to air puffs from the lateral side^26^. Here, we compared walking trajectories of the initial responses to the lateral puff among the three stimulation types.

**Figure 3:**
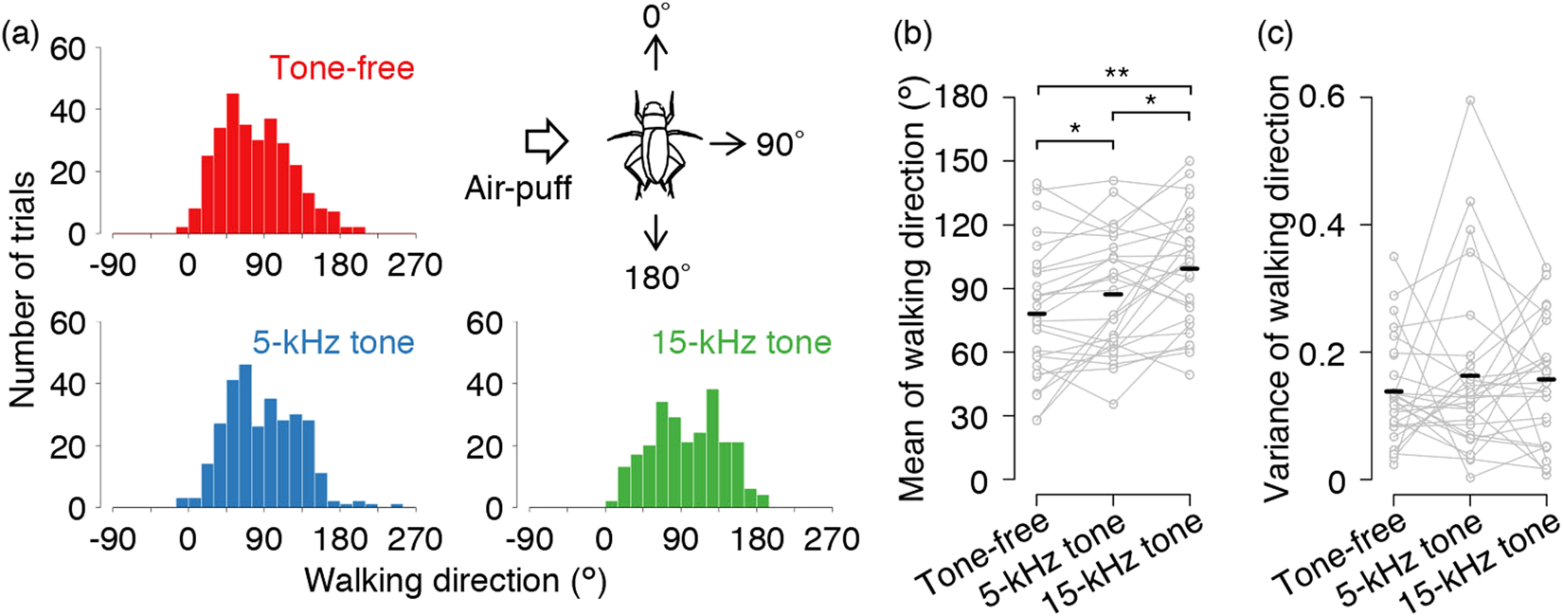
Auditory effects of different frequencies of tones on directionality in wind-elicited escape. (**a**) Histograms of walking direction for three types of stimulation in 15-degree bins (n = 299 trials for tone-free, n = 299 trials for 5-kHz tone, and n = 250 trials for 15-kHz tone). Upper right inset indicates a definition of the walking direction. Direction of walking opposite to the stimulus angle was defined as 90º, and walking forward and backward resulted in 0º and 180º, respectively. (**b**,**c**) Mean angle and circular variance of walking direction in response to three types of stimulation. (N = 27 individuals) **p < 0.01, *p < 0.05 (paired t-test followed by Holm’s correction).

Typical walking trajectories in response to lateral stimulus showed that a cricket walked backward more frequently after 15-kHz tone stimulation than after other frequency (Fig. 1). Frequency distributions of the walking direction revealed that the 15-kHz stimulation was different at its peak from the tone-free and 5-kHz stimulation (Fig. 3a). Comparing the walking direction for individuals among the three types of stimulation, both frequencies of tones facilitated walking in the backward direction (*p* = 0.0002, one-way repeated-measures ANOVA, *p* = 0.0241 for tone-free vs 5-kHz tone, *p* = 0.0032 for tone-free vs 15-kHz tone, Holm’s corrected paired t-test) (Fig. 3b). In addition, the 15-kHz tone facilitated backward movement to a larger degree than the 5-kHz tone (*p* = 0.0241 for 5-kHz tone vs 15-kHz tone). This suggests that the higher frequency tone has a larger impact on walking direction. To determine whether these auditory modulations on walking direction were derived from the inhibition of forward walking, we compared the dispersion of the frequency distributions and the individual changes of circular variance of walking direction among the three stimulation types. We observed no significant differences in both frequency distribution of the population (*p* = 1 for all pairs, Wallraff’s test followed by Holm’s correction) and circular variance for each individual (*p* = 0.6050, one-way repeated-measures ANOVA) (Fig. 3a and c). These results demonstrated that the tones did not simply inhibit forward walking but also induced walking in the backward direction more frequently.

### Frequency dependency of the auditory modulation of turn magnitude

In the wind-elicited escape, the walking direction linearly correlates with the stimulus angle, meaning that the crickets always move in the direction opposite to the air puff^24^. In contrast, the turn angle depends on the stimulus angle according to a sin function because backward motion can consist of one of two movement strategies, one of which is turnaround followed by straight walking, and the other is backward stepping without turn^27,28^. Here, to examine which strategy of walking in the backward direction was enhanced by tones, we analysed the cross-modal effects on the absolute turn angle (termed ‘turn magnitude’) and their frequency dependency.

**Figure 4:**
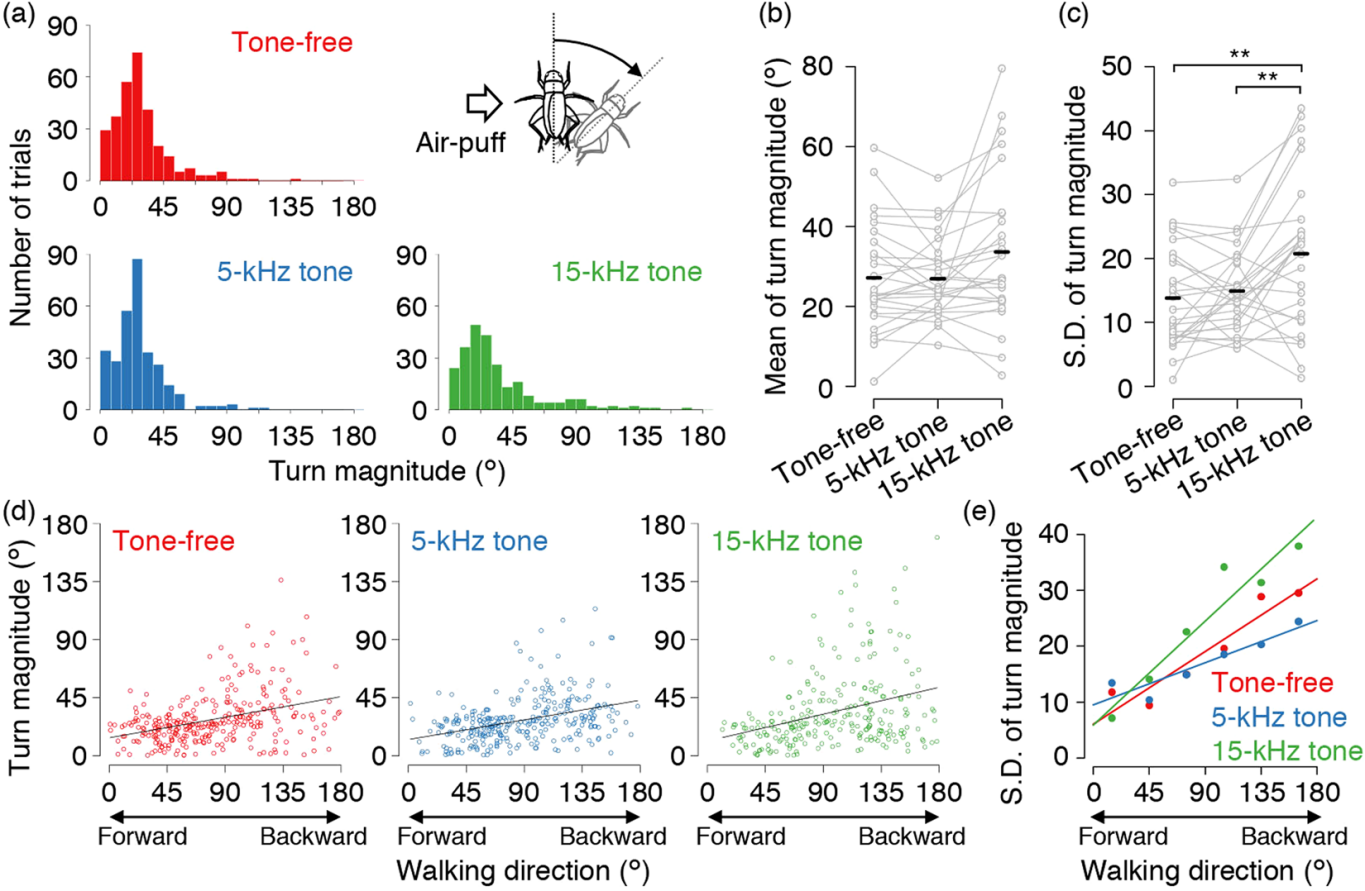
Auditory effects of different frequencies of tones on turn movement in wind-elicited escape. (**a**) Histograms of turn magnitude for three types of stimulation in 7.5-degree bins. Upper right inset indicates a definition of turn magnitude. The turn magnitude was measured as an absolute angular value of turn regardless of the rotational direction. (**b**,**c**) Mean and standard deviation of turn magnitude among the three types of stimulation. (**d**) Correlations between walking direction and turn magnitude for the tone-free (r = 0.3729, *p* < 0.0001), 5-kHz tone (r = 0.3787, *p* < 0.0001), and 15-kHz tone (r = 0.3273, *p* < 0.0001) stimulations. Unlike the aligned walking direction shown in Fig. 3, the walking direction was measured as an absolute value in which forward and backward walks resulted in 0º and 180º, respectively. **p < 0.01, (paired t-test followed by Holm’s correction). (**e**) Correlations between walking direction and standard deviation of turn magnitude calculated in 6 bins equally-spaced per 30º (0º–30º, 30º–60º, 60º–90º, 90º–120º, 120º–150º, and 150º–180º). The standard deviation was positively correlated with walking direction in all types of stimulation (for tone-free, r = 0.9442, *p* = 0.0046; for 5-kHz tone, r = 0.9282, *p* = 0.0075; for 15-kHz tone, r = 0.9584, *p* = 0.0026).

The frequency distributions of the turn magnitudes were similar to each other at their peaks among the three stimulation types (Fig. 4a). There was no significant difference in mean turn magnitude among the three stimulation types (*p* = 0.0073, one-way repeated-measures ANOVA, *p* = 0.6436 for tone-free vs 5-kHz tone, *p* = 0.0574 for tone-free vs 15-kHz tone, *p* = 0.0574 for 5-kHz tone vs 15-kHz tone, Holm’s corrected paired t-test) (Fig. 4b). Focusing on the variance of the distributions, however, the turn magnitude after 15-kHz tone stimulation showed broader distribution than those of tone-free or 5-kHz tone stimulation (*p* < 0.0001 for tone-free vs 15-kHz tone, *p* < 0.0001 for 5-kHz tone vs 15-kHz tone, *p* = 0.0901 for tone-free vs 5-kHz tone, F test followed by Holm’s correction). This indicates that the 15-kHz tone induced larger turns more frequently. For each individual, the turn magnitude fluctuated more after the 15-kHz tone stimulation than after tone-free or 5-kHz tone stimulation (*p* < 0.0001, one-way repeated-measures ANOVA, *p* = 0.0010 for tone-free vs 15-kHz tone, *p* = 0.0055 for 5-kHz tone vs 15-kHz tone, *p* = 0.3191 for tone-free vs 5-kHz tone, Holm’s corrected paired t-test) (Fig. 4c). Taken together, it is suggested that the 15-kHz tone causes large turns, resulting in broader distribution of the turn magnitude.

The 15-kHz tone facilitated walking in the backward direction (Fig. 3b) and increased the variance of the turn magnitude. Were these effects of the 15-kHz tone independent from each other? To answer this question, we examined the relationships between the walking direction and the turn magnitude. In all types of stimulation, the turn magnitude was positively dependent on the walking direction, but there was no significant cross-modal effect on that dependency (*p* = 0.2565 for tone-free vs 5-kHz tone, *p* = 0.0971 for tone-free vs 15-kHz tone, multiple regression analysis) (Fig. 4d). This means that the more backward the crickets moved, the more largely they turned regardless of sound. The Pearson’s correlation coefficients for these relationships, however, were low because more walking in the backward direction was accompanied by a more varied turn magnitude. In addition, the scatter plots shown in Fig. 4d indicate that large turns were more common after the 15-kHz tone stimulation than after tone-free or 5-kHz tone stimulation. Further, we examined the relationships between the walking direction and the standard deviation of turn magnitude (Fig. 4e, see also Methods). The standard deviation of the turn magnitude was strongly correlated with the walking direction for all types of stimulation (Fig. 4e). Interestingly, the slope of regression line for the 15-kHz tone was significantly larger than that of tone-free (*p* = 0.0027, multiple regression analysis), whereas there was no significant difference between the 5-kHz tone and tone-free (*p* = 0.0813) (Fig. 4e). Therefore, the high frequency tone facilitated the walking backward, and additionally caused fluctuations in turning movement.

## Discussion

In this study, we found that the sound modulates wind-elicited escape behaviour in the response probability, walking distance, walking direction, and variance of turn magnitude. These effects would result from cross-modal interaction between the auditory system and cercal system, because we removed antennae from the tested crickets, which are also the other mechanosensory organ which may detect the airflow from front. These cross-modal effects depended on the sound carrier frequency of the stimuli. Before the experiments, we had predicted that the 5- and 15-kHz tones would have different impacts on the wind-elicited escape because these frequencies of sounds directly trigger different behaviours in the flying cricket, which are positive and negative phonotaxis^13,14^. Based on these facts, we hypothesized that crickets can recognise different contexts based on sound frequency. Reduction of the response probability, elongation of the walking distance, and enhancement of the turn-angle variability were exerted by 15-kHz but not 5-kHz tones. Although the 5-kHz tone facilitated walking in the backward direction similarly to the 15-kHz tone, this effect of the 5-kHz tone was significantly smaller than that of the 15-kHz tone. These results reveal that crickets alter their wind-elicited escape strategies depending on the acoustic context.

Even innate behaviours of insects are not only simple reflex responses or stimulus-triggered hardwired behaviours, but are adaptively modulated depending on the environmental contexts and physiological states, which are mediated by cross-modal interaction between the different sensory systems. Crickets perceiving a ‘shelter’ alter their wind-elicited escape behaviours so that the crickets turn toward the shelter^29^. The fruit fly reduces CO_2_ avoidance behaviour in the context of appetitive odour^30^. Our previous study also demonstrated that crickets alter their wind-elicited escape behaviour in moving direction and response threshold after hearing an acoustic stimulus^26^. This suggests that insects’ avoidance behaviours can be flexibly modulated by various sensory inputs including visual, olfactory, and auditory signals. Furthermore, other innate behaviours also depend on the sensory parameters representing a context. For example, the male fruit fly modulates the intensity of the calling song to attract the conspecific female depending on the visually estimated distance to the female fruit fly^31^. Our present results demonstrating the sound-frequency dependence of the auditory modulation support the idea that the insect auditory system, which had been considered as simply triggering the specific behaviours^7,8^, is also used for perception of the acoustic context instructing decision making.

The 15-kHz tone facilitated walking in the backward direction and reduced response probability of wind-elicited escape, which corresponds to our previous study^26^. In addition to these effects, the walking distance was elongated by the 15-kHz sound. In general, a longer escape distance results in a higher survival rate from attacking predators^32^. In contrast, the decrease in the response probability of walking means that the crickets less frequently response to the air-puff stimuli, which is also one of their escape strategies^33,34^. In the context of the presence of echolocating bats, which are a predator of the crickets, an inadvertent motion in response to weak air-flows may result in being caught by predators. Indeed, gleaning bats could potentially detect movement of the cricket on the ground by passive hearing and locate to capture it^35^, while the standing cricket exhibits no response to the ultrasound emitted by bats^22^. The elevation of the response threshold would be, therefore, effective to avoid the detection by bat’s echolocation call. The ethological meaning of the facilitation of walking backward with a 15-kHz tone remains unknown. The walking in the backward direction that is an unanticipated movement contrary to the bat’s expectation may be also one of the effective escape strategies.

Another effect specific to the 15-kHz tone was increase in variance of the turn magnitude. The turn magnitude measured in this study indicates the orientation to which the cricket faced at the termination of initial response^24,26^. Although the walking direction was altered by the sound, its variance was unaffected, meaning that the auditory inputs make no change in the directional variability of the initial responses. In the context of the presence of predators, therefore, crickets may utilize their second responses to further move in various directions. For the behavioural analysis in this study, we focused on the initial response but paid no attention to the second or later responses because few second responses were observed in this study (data were not shown). It is possible that repetitive air-puffs may uncover the directional variability of second or later responses^36^.

Variability in the escape behaviour is thought to be not only noise but also an adaptive trait, which leads to unpredictability for protection from predators^37–39^. Some previous studies on fish demonstrate that the escape direction was varied depending on the context^40^. For example, acute changes in environmental conditions, such as hypoxia and cooling, facilitate the movement of fish toward the noxious stimulus instead of away from it, resulting in greater variability of escape direction^41,42^. In addition, herring schools exhibit escape responses away from the stimulus more frequently than a solitary fish^43^. These facts suggest that animals alter the variability in the escape behaviours depending on the surrounding contexts. Despite the significance of these findings, the neural mechanism producing the behavioural variability remains unknown. An insect’s escape behaviour being regulated by a small neural circuit consisting of a few accessible, identified neurons is an appealing behavioural model^44^. The cross-modal effect on the behavioural variability in the cricket escape behaviour will be useful to understand the neural mechanisms that regulate the behavioural variability.

The 5-kHz tone mainly activating AN1, which encodes the calling song pattern, may mean the presence of a conspecific male, while the 15-kHz tone dominantly activating AN2, which directly triggers avoidance response during flight, may be perceived as an alarm signal against an approaching bat^2^. The larger impacts of the 15-kHz tone over the 5-kHz tone suggest that AN2 plays a greater role in the acoustic modulation of the wind-elicited escape behaviour. On the other hand, cercal giant interneurons (GIs) respond to the air-puff stimuli but not to high-frequency sound like 5- or 15-kHz pure tones, and some types of GIs contribute directly to escape walking^24,45^. All GIs convey the cercal sensory information from the terminal abdominal ganglion to the thoracic ganglia and the brain (reviewed by ref.^46^). Within the brain, GIs arborize mainly in the dorsal region of the deutocerebrum^47^, where AN2’s ascending axon is terminated^16,17^. These adjacent projections imply that the auditory and cercal sensory information may be integrated at this region, resulting in the auditory modulations of wind-elicited walking. There is no evidence of synaptic inputs from auditory receptors or interneurons onto GIs within the prothoracic ganglion. However, it is still possible that the cercal ascending signals are modulated by auditory inputs at the early sensory processing stage. Clarifying the integration regions and neural circuits involved in the cross-modal modulations is a major question for future studies.

## Methods

Methods for behavioural experiments, stimulation, and data analysis are similar to those used in our previous studies^24,26^.

### Animals

Laboratory-bred adult male crickets (*Gryllus bimaculatus*, De Geer 1773) (0.50–0.80 g body weight) were used throughout the experiments. They were reared under 12:12-h light-dark conditions at a constant temperature of 27ºC. The guidelines of the Institutional Animal Care and Use Committee of the National University Corporation, Hokkaido University, Japan, specify no requirements for the treatment of insects in experiments. Their antennae were removed just before behavioural tests to eliminate the influence of mechanosensory inputs from the antennal organ so we could focus on the interaction between cercal and auditory systems.

### Treadmill system

Locomotion of crickets was monitored with an open-loop spherical-treadmill system, which was used in our previous studies^24,26,36^. A cricket was tethered on the top of Styrofoam ball with a pair of insect pins bent into an L-shape that were fastened to the cricket’s tergite with paraffin wax. The ball rotation was monitored at a 200-Hz sampling rate using two optical mice mounted orthogonally around the ball. TrackTaro software (Chinou jouhou shisutemu, Kyoto, Japan) was used to measure the virtual walking trajectory and to calculate translational and angular turn velocities based on the measured ball rotation.

### Air-puff and acoustic stimulations

An air-puff stimulus was applied to the stationary cricket by a short puff of nitrogen gas from a plastic nozzle (15 mm diameter) connected to a PV820 pneumatic picopump (World Precision Instruments, Sarasota, FL, USA). The nozzle ends were arranged at a 105-mm distance from the animal. The air-puff velocity used was 0.90 m/s measured at the centre of the treadmill ball with a thermal anemometer (405-V1, Testo, Yokohama, Japan) and regulated by adjusting the delivery pressure of the picopump. This stimulus velocity we used was much faster than the air currents produced by the approaching predators^25^, and effective to elicit escape walking modulated by the sound stimulus in our previous study^26^.

The acoustic stimuli of 5- or 15-kHz pure tone were synthesized using RPvdsEx software (Tacker Davis Technologies, Alachua, FL, USA), and transduced and attenuated using a RM1 processor (TDT). The sounds were delivered by 1.5-inch (3.81 cm) full-range sealed MM-SPS2 loudspeakers (Sanwa Supply, Okayama, Japan). The sound pressure is calibrated at 70 dB SPL at the centre of the treadmill ball with a sound-level meter (TYPE 6224, ACO CO., LTD., Tokyo, Japan). The speakers were located just above the air-puff nozzles. To avoid a sound reverberation, anechoic foam was attached to the inside wall of the recording chamber. All the experiments were conducted in a sound-proof chamber with a 150-mm-thick wooden wall.

### Experimental procedure

To test dependency of sound carrier frequency of the auditory effects on wind-elicited escape, we used three types of stimulation referred to as tone-free, 5-kHz tone, and 15-kHz tone (Fig. 1). For the cross-modal stimulation, 5- or 15-kHz tone sound of 1-s duration was initiated 800 ms before an air-puff for 200 ms. For the uni-modal stimulation referred to as tone-free, a 200-ms air-puff was delivered without any prior acoustic stimulus, but the cricket’s walking activities were monitored during the 800-ms silent time prior to the air-puff. In all types of stimulation in this study, a sequence of stimuli was started only if the cricket remained at rest for 1 s or longer. The acoustic and air-current stimuli were always delivered from the same direction as shown by inset of Fig. 1.

Each individual cricket was randomly exposed to three types of stimulation for 20 trials each, leading to 60 trials in total. The inter-trial interval was >1 minute. Each cricket received all stimuli from a single direction, with 14 crickets receiving from the left side and 13 crickets from the right side. For the data analysis, we combined the results of the groups stimulated by left and right sides, therefore, the sample size for the number of crickets were 27.

### Data analysis

Behavioural data provided by the TrackTaro software were analysed off-line, using custom algorithms with R programming software (version 3.3.3, R Development Core Team). We classified the recorded data of all trials (n = 1620) into three responses referred to as ‘wind-elicited response’, ‘sound-elicited response’, or ‘no response’ based on the walking speed, as used in previous work^26^. The auditory response probability was defined as follows:

1 $${\rm{Auditory}}\,{\rm{response}}\,{\rm{probability}}=\frac{{N}_{s}}{{N}_{s}+{N}_{w}+{N}_{no}}$$

where ${N}_{s}$, ${N}_{w}$, and ${N}_{no}$ are the number of trials categorized as a ‘sound-elicited’ response, a ‘wind elicited’ response, and a ‘no response’, respectively^26^. Similar to our previous findings, we confirmed that neither 5- nor 15-kHz tones changed the ‘auditory response probability’, meaning that both frequencies of sound rarely elicited walking reactions (Fig. S1). In further analyses, therefore, we eliminated the trials categorized into ‘sound-elicited response’ and focused on ‘wind-elicited’ responses (n = 848). The wind-elicited response probability was defined as follows:

2 $${\rm{Wind}}\,{\rm{response}}\,{\rm{probability}}=\frac{{N}_{w}}{{N}_{w}+{N}_{no}}$$

We measured the following locomotor parameters in the ‘initial response’ of the wind-elicited walking, which was defined as a continuous walking trot followed by a stationary moment^24,26^: walking distance, reaction time, maximum walking speed, walking direction, and turn magnitude. The walking direction was defined for forward as 0º so that the direction opposite to stimulus angle was 90º and backward direction was 180º (see insets in Fig. 3a). The turn magnitude was measured as an absolute value to compare the magnitude of turning movement. In Fig. 4d and e, we used absolute walking direction defined as follows: 0º means the cricket moved in the forward direction and 180º means the cricket moved in the backward direction. For statistical analyses of the scalar parameters such as reaction time, maximum walking speed, walking distance, and turn magnitude, we calculated average values in trials categorized into ‘wind-elicited response’ for each individual to avoid pseudo-replication. For analysis of the walking direction that was a circular parameter, we calculated mean of angle and circular variance for each individual using a package in R programming for circular statistics^48^. Prior to the statistical test of significance of the stimulation types, we checked the distribution of all dataset for mean and S.D. values for each individual, using Kolmogorov-Smirnov test. To assess the significance of the stimulation types for response probability (Fig. 2a), maximum walking speed (Fig. 2c), reaction time (Fig. 2d), walking direction (Fig. 3b and c), and turn magnitude (Fig. 4b and c), of which the data were distributed in Gaussian, we used one-way repeated-measures analysis of variance (ANOVA). If the main effect of stimulation types was significant, we used a paired t-test followed by Holm’s correction as a *post hoc* test. To assess the significance of the stimulation types for walking distance (Fig. 2b) and auditory response probability (Fig. S1), of which the data were not distributed in Gaussian, we used Friedman’s test and Wilcoxon paired-sample test instead of ANOVA and t-test, because the data sets were different from Gaussian distribution. To compare the variance in distributions of the walking direction among the three types of stimulation, we used Wallraff’s test using the R programming package of circular statistics^48,49^. To compare the variance in distributions of the turn magnitude among the stimulation types, we used an F test. To assess the significance of sound frequency for the relationships between the walking direction and the turn magnitude, we used a multiple regression analysis for their plots in absolute values considering an interaction effect of the walking direction and the stimulation type. For analysis of the correlation between the walking direction and the variance of turn magnitude, we calculated standard deviations of the turn magnitude for ranges of every 30º (i.e. 0º–30º, 30º–60º, 60º–90º, 90º–120º, 120º–150º, and 150º–180º) of the walking direction. Further, we tested the significance of the stimulation type for that relationship, using multiple regression analysis.

## Electronic supplementary material

Figure S1
