# Supplementary material for: Crickets alter wind-elicited escape strategies depending on acoustic context
Source: Sci Rep. 2017 Nov 9;7:15158. doi: 10.1038/s41598-017-15276-x (PMC5680309; doi:10.1038/s41598-017-15276-x)
Supplement: Supplementary file 1 — Figure S1 [file 41598_2017_15276_MOESM1_ESM.pdf]

Crickets alter wind-elicited escape strategies depending on acoustic context

Matasaburo Fukutomi and Hiroto Ogawa

Supplementary Figure

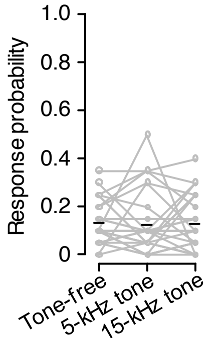

Figure S1. Probability of walking initiation for the period of 800 ms before the onset of an air-puff. There was no significant difference in the response probabilities among the three types of stimulation ($p = 0.3679$, Friedman's test), meaning that an auditory stimulus of 5- or 15-kHz frequency triggered no walking in crickets on its own.
